# CoLeaf-DB: Peruvian coffee leaf images dataset for coffee leaf nutritional deficiencies detection and classification

**DOI:** 10.1016/j.dib.2023.109226

**Published:** 2023-05-12

**Authors:** Victor A. Tuesta-Monteza, Heber I. Mejia-Cabrera, Juan Arcila-Diaz

**Affiliations:** Facultad de Ingeniería Arquitectura y Urbanismo, Universidad Señor de Sipán, Perú

**Keywords:** Peruvian coffee, Image datasets, Machine learning, Deep learning, Deficiencies detection

## Abstract

This article introduces Peruvian coffee leaf datasets known as CATIMOR, CATURRA and BORBON of coffee plantations located at San Miguel de las Naranjas and La Palma Central, Jaén province, Cajamarca, Perú. The leaves with nutritional deficiencies were identified by agronomists, using a physical structure the controlled environment was designed and the images were captured with a digital camera. The dataset contains 1006 leaf images grouped according to their nutritional deficiencies (Boron, Iron, Potasium, Calcium, Magnesium, Manganese, Nitrogen and others). CoLeaf dataset contain images that facilitate training and validation during the utilization of deep learning algorithms for coffee plant leaf nutritional deficiencies recognition and classification. The dataset is publicly and freely available at http://dx.doi.org/10.17632/brfgw46wzb.1.

**Specifications Table**

**Table:** 

Subject	Computer Science, Agricultural Science, Biological Science
Specific subject area	Artificial Intelligence, Computer Vision and Pattern Recognition
Type of data	Images
How the data were acquired	The coffee leaves were collected and classified according to nutritional deficiencies by an agronomist engineer. Raw JPEG images of the leaves of coffee plants were captured under controlled environment with a Canon PowerShot SX50 HS camera with a sensor CMOS, resolution of 12.1MP.
Data format	Raw, the data are in jpeg format
Description of data collection	The images present in the dataset were captured in a controlled environment. The physical structure of the controlled environment allowed to control the distance from the digital camera to the object (coffee leaf), improve luminosity, distribute the light and to have a 0% shadow capture.
Data source location	Source location was coffee plantations located at San Miguel de las Naranjas and La Palma Central, Jaén province, Cajamarca, Perú with a Latitude 5° 44′ 27.2" S and a longitude of 78° 51′ 25.4" W, in the year 2014.
Data accessibility	The datasets are publicly and freely available on mendeley data repository with DOI: 10.17632/brfgw46wzb.1 at http://dx.doi.org/10.17632/brfgw46wzb.1
Related research article	Vassallo-Barco M., Vives-Garnique L., Tuesta-Monteza V., Mejía-Cabrera H.I., Toledo R.Y., Automatic detection of nutritional deficiencies in coffee tree leaves through shape and texture descriptors, Journal of Digital Information Management, 15 (1) (2017) 7-18.

## Value of the Data

• Data is used for evaluating algorithms, which are used in machine learning or deep learning for training, testing and validation of classification of nutritional deficiencies for instance Nitrogen, Phosphorus, Potassium, Magnesium, Boron, Calcium, Manganese, Calcium, Iron and others.
• This dataset encourages and motivates further research of machine learning about the characteristics of coffee leaves.
• Nutritional deficiencies in coffee plants affect production and therefore it is important its early identification. This dataset can be used in improving the accuracy of coffee leaf nutritional deficiencies detection and classification.
• In addition to deep learning the present dataset can be used for the extraction of specific characteristics of coffee leaves, such as shape, texture, color, to analyze images, as well as for segmentation tasks. It is also possible to employ classical machine learning such as SVM, decision trees, specific rule-based image processing to identify nutritional deficiencies in coffee leaves.

## 1 Objective

Leaves analysis for evaluating the nutritional state of the crops is a practice commonly used [1], by analyzing coffee leaves, nutritional deficiencies [2] and common diseases [3,4] can be determined. The present dataset has been used to automatically detect nutritional deficiencies considering the shape and color of coffee leaves, using a naive Bayes classifier and another classifier based on neural networks, obtaining for both results that can be improved using other classification and processing methods.

## 2 Data Description

The composition of the Coleaf-DB data set organized in Table 1 is shown below. This dataset is composed of 10 folders representing the nutritional deficiency of Nitrogen (N), Phosphorus (P), Potassium (K), Magnesium (Mg), Boron (B), Manganese (Mn), Calcium (Ca), Iron (Fe), healthy leaves and leaves with more than one deficiency present in the leaves of the coffee tree. The images within each folder have the annotations of the nutritional deficiency in their respective file name. The second column of Table 1 indicates the number of images contained in each folder, with dimensions of 3000×4000 px in compressed jpeg format with a horizontal and vertical resolution of 180 dpi and a depth of 24 bits.

**Table 1:** Number of digital images by nutritional deficiency of coffee leaves from the Coleaf-DB dataset.

Nutritional deficiencies of Leaf	Number of images
Healthy leaf	6
Nitrogen (N)	64
Phosphorus (P)	246
Potassium (K)	96
Magnesium (Mg)	79
Boron (B)	101
Manganese (Mn)	83
Calcium (Ca)	162
Iron (Fe)	65
More than one deficiency	104

**Fig 1:**
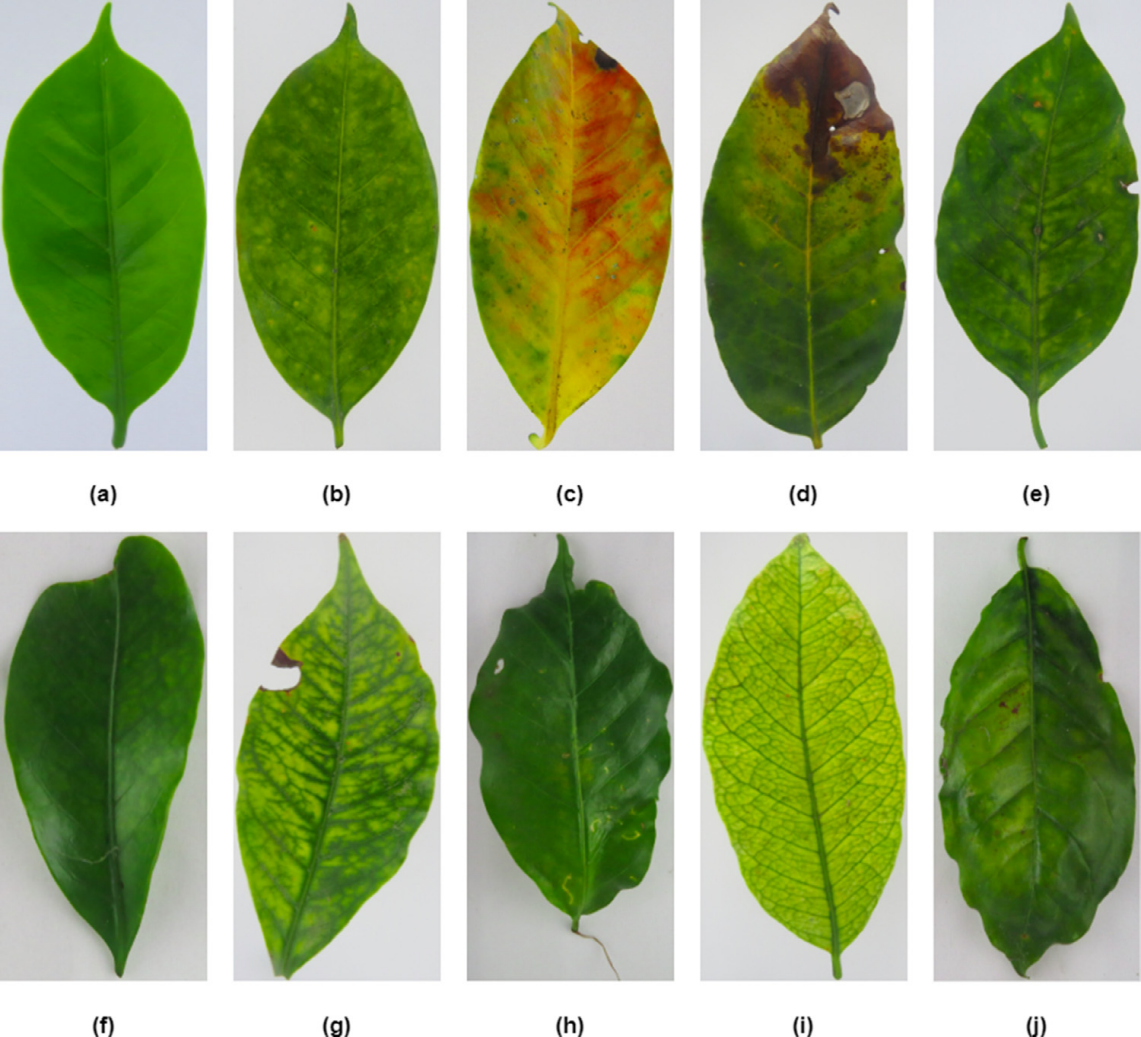
Healthy leaf (a), Nitrogen deficiency (b), Phosphorus deficiency (c), Potassium deficiency (d), Magnesium deficiency (e), Boron deficiency (f), Manganese deficiency (g), Calcium deficiency (h), Iron deficiency (i), Leaves with more than one deficiency (j).

The researchers conducted a tour of the coffee plantation to collect leaves of the varieties CATIMOR, CATURRA and BORBON in their second productive stage, after the phenological phase of maturation after having harvested the first production and waiting for the next phase of flowering, about 4 years after planting. In a controlled environment, images were taken and classified according to nutritional deficiency considering leaf characteristics (interveinal coloration, chlorosis and deformation). This classification was carried out by an agronomist engineer using the observation method. Fig. 1(a) below shows the image of a healthy leaf.

Nutritional deficiencies and their representative characteristics are described below.

### 2.1 Nitrogen (N)

The symptoms appear first in the old or developed leaves and progress towards the young parts, presenting a uniform chlorosis that advances from the base towards the apex of the leaf and from the central vein towards the edges of the leaf, when the deficiency is more severe it becomes more chlorotic and covers the entire leaf blade. A total of 64 images were taken and processed with this nutritional deficiency. Fig. 1(b) below shows the image of a leaf with Nitrogen deficiency.

### 2.2 Phosphorus (P)

Older leaves show lobular interveinal chlorosis, with irregularly shaped yellow-tan spots showing areas with a reddish hue. A total of 246 images were taken and processed with this nutritional deficiency. Fig. 1(c) below shows the image of a leaf with Phosphorus deficiency.

### 2.3 Potassium (K)

At the beginning, a yellow-brown spot appears as a band, becoming necrotic later with a dark brown shade, a yellow halo limits the necrosis of the edge; this deficiency appears first in the old leaves, finally the edges of the leaves and the tips curl upwards [5]. A total of 96 images were taken and processed with this nutritional deficiency. Fig. 1(d) below shows the image of a leaf with Potassium deficiency.

### 2.4 Magnesium (Mg)

This nutritional deficiency is shown by interveinal chlorosis on the older leaves of the plant. The yellowing starts at the base of the branch and spreads towards the tip, with green stripes appearing along the midrib of the leaf forming an inverted wedge towards the petiole, the chlorosis is followed by rapid and severe defoliation. A total of 79 images were taken and processed with this nutritional deficiency. Fig. 1(e) below shows the image of a leaf with Magnesium deficiency.

### 2.5 Boron (B)

This deficiency manifests itself in the young leaves, which are small, elongated, twisted, wrinkled, with irregular edges, deformed and leathery in texture. Leaves show a dull olive-green chlorosis extending from the apex to the base, the leaf margin is rough. Older leaves are yellowish at the tip, showing suberization and the midrib and secondary veins are corky. A total of 101 images were taken and processed with this nutritional deficiency. Fig. 1(f) below shows the image of a leaf with Boron deficiency.

### 2.6 Manganese (Mn)

This deficiency shows up in the young leaves with a pale green color, with the main veins and a band on either side remaining deep green. As the deficiency progresses, the leaves turn increasingly yellow. A total of 83 images were taken and processed with this nutritional deficiency. Fig. 1(g) below shows the image of a leaf with Manganese deficiency.

### 2.7 Calcium (Ca)

This deficiency is shown by marginal chlorosis of new leaves. Chlorosis is regularly associated with a deformation of the leaf which acquires a convex shape and with the formation of cork in the veins on the underside of the leaves. Leaves lose their erect condition and hang downward without abscission. A total of 162 images were taken and processed with this nutritional deficiency. Fig. 1(h) below shows the image of a leaf with Calcium deficiency.

### 2.8 Iron (Fe)

In plants exhibiting Fe deficiencies the new leaves take on colorations ranging from greenish yellow to very light green (almost white), while the veins remain green, forming a very fine netting [6]. A total of 65 images were taken and processed with this nutritional deficiency. Fig. 1(i) below shows the image of a leaf with Iron deficiency (Table 2).

**Table 2:** Color and shape characteristics of leaves with nutritional deficiencies.

Nutritional deficiency	Color characteristic	Shape characteristic
Nitrogen (N)	Light yellow starting at the base.	
Phosphorus (P)	Red and brown (burn).	
Potassium (K)	Brown (burn) with neighboring yellow areas at the apex.	
Magnesium (Mg)	Very light green in the leaf area (between the veins), with green veins.	
Boron (B)		Rounded apex, rounded leaf shape.
Manganese (Mn)	Light green in the leaf area with loss of the tertiary veins, the main vein maintains the green color.	
Calcium (Ca)		Ribbed leaf shape.
Iron (Fe)	Light yellow leaf, with well-defined veins.	

## 3 Experimental Design, Materials and Methods

### 3.1 Data Acquisition

The number of samples for each type of nutritional deficiency was limited by the manifestations of the plantations visited in San Miguel de las Naranjas and La Palma Central in the province of Jaen in Peru, which are located approximately 1459 meters above mean sea level, with a temperature between 11°C and 25°C and an annual rainfall between 700mm and 2000mm approximately. A possible bias is presented in the imbalance of samples for each nutritional deficiency, because the state of health of the plantations has depended on the agricultural management of the coffee plant by the owners.

In the area of sample acquisition, it was found that the plants showed recurrent manifestations of 8 nutritional deficiencies, these being Nitrogen (N), Phosphorus (P), Potassium (K), Magnesium (Mg), Boron (B), Manganese (Mn), Calcium (Ca), Iron (Fe), likewise, the number of samples for each deficiency is related to the field observations by the agronomist during the visits, carrying out the following tasks:

a) The work team led by the agronomist identified the area of land with the coffee plantation and was located in one of the corners where the plantation began with the purpose of making a sweep in an orderly manner.
b) Each one of the plants was checked to collect samples of leaves with nutritional deficiencies identified according to what was found, for this reason the quantities were not balanced.
c) In each plant the agronomist made a thorough observation and took the leaf that adequately represented the nutritional deficiency, verifying with the table of characteristics of nutritional deficiencies, then the corresponding classification was made.

Images were captured in a controlled environment using a Canon PowerShot SX50 HS digital camera with a CMOS sensor and 12.1MP resolution.

**Fig 2:**
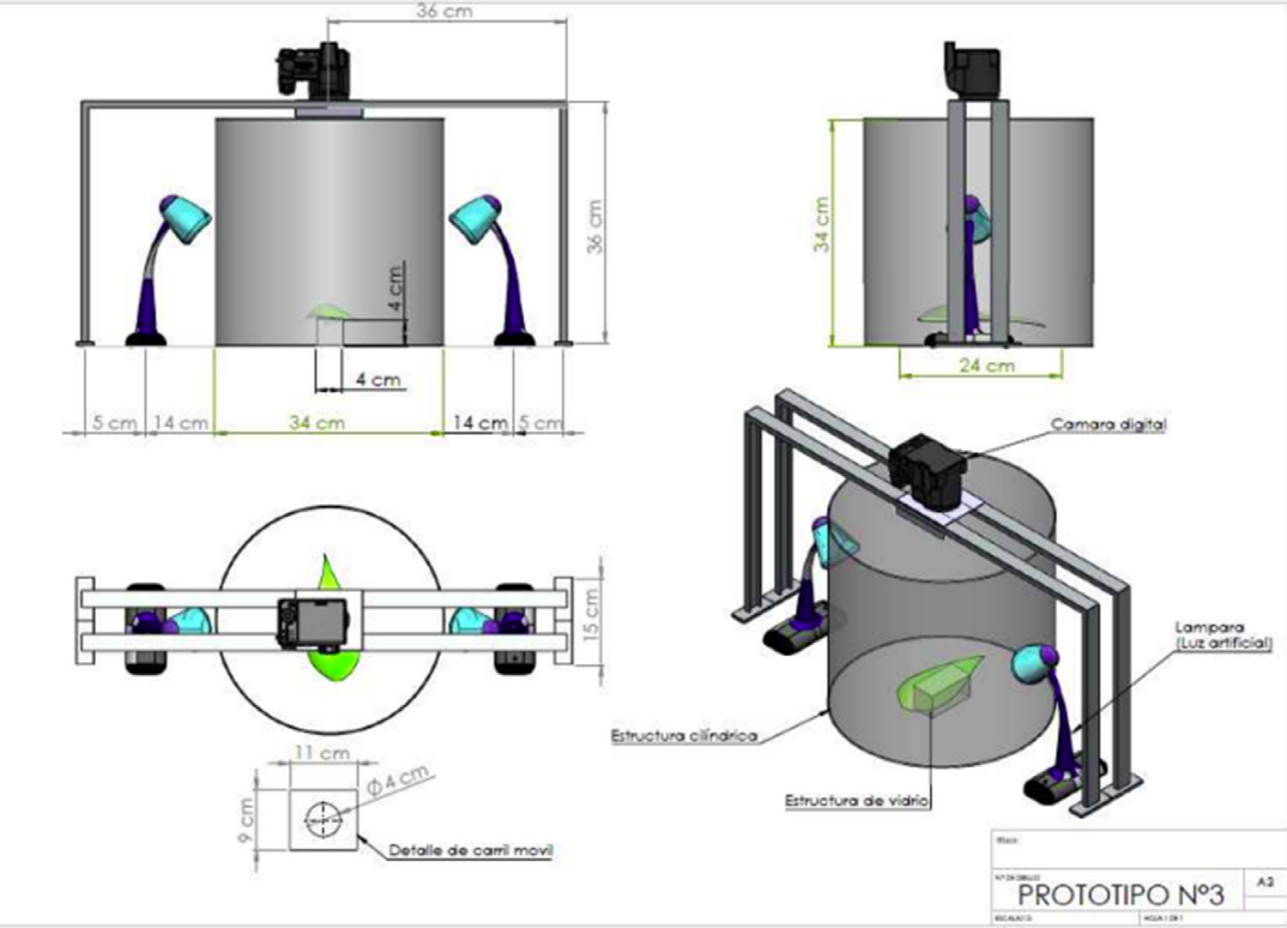
Physical structure of the controlled environment for image capture.

Considering the recommendations of the photographic experts, the physical structure of the controlled environment for image capture was built (Fig. 2). This physical aluminum structure allowed to control the top, bottom, right and left distance from the digital camera to the object (coffee leaf). To control the light intensity, a cylindrical structure of 34cm in diameter and 34cm in height was built, surrounded by white muslin cloth; this cloth was chosen because it allows the passage of light in an adequate manner. Two 250 lumen artificial light sources were included, placed at a distance of 14 cm each from the cylindrical structure and at about 45 degrees of light beam angle with respect to the object. The capture of images is done at a distance of 34cm, which allows capturing complete leaves of different sizes, the luminosity was improved without bouncing due to the cylindrical structure that evenly distributes artificial and natural light, also to have a capture with 0% shadows a transparent stool was placed that allows having the object (coffee leaf) suspended in the air. The images acquired with dimensions of 3000×4000 pixels were not preprocessed in order to preserve all the information in them, so that the researchers can use the dataset with the original data and apply the preprocessing techniques according to their research objectives.

### 3.2 Preliminary Results

To obtain preliminary results, the deep convolutional neural network ResNet50 was used to classify coffee nutritional deficiencies in the Coleaf-DB dataset. The dataset was divided into a training set with 800 images and a test set with 200 images, i.e. from each group of nutritional deficiencies 80% of images have been taken for training and 20% for evaluating the neural network. The images were resized to 224×224 pixels to fit the ResNet50 input, normalizing all pixels.

The model was trained for 100 epochs with a batch size of 64 and a learning rate of 0.001. The cross-entropy loss function and the Adam optimizer were used. The model achieved an accuracy of 87.75% on the test set.

## Ethics Statements

The dataset presented in this work does not include tests on animals or humans. All images used were obtained by the authors and do not come from any other source.

## CRediT authorship contribution statement

**Victor A. Tuesta-Monteza:** Writing – review & editing, Investigation. **Heber I. Mejia-Cabrera:** Writing – original draft. **Juan Arcila-Diaz:** Methodology, Conceptualization.

## Declaration of Competing Interest

The authors declare that they have no known competing financial interests or personal relationships that could have appeared to influence the work reported in this paper.

## Data Availability

CoLeaf DATASET (Original data) (Mendeley Data).
